# Fluorescence Detection and Discrimination of ss- and ds-DNA with a Water Soluble Oligopyrene Derivative

**DOI:** 10.3390/s90604164

**Published:** 2009-06-02

**Authors:** Youqiang Chen, Gaoquan Shi

**Affiliations:** Key Lab of Bio-organic Phosphorous Chemistry and Chemical Biology, Department of Chemistry, Tsinghua University, Beijing 100084, China; E-Mail: chen-yq06@mails.tsinghua.edu.cn (Y.C.)

**Keywords:** water-soluble oligopyrene derivative, DNA, fluorescence quenching

## Abstract

A novel water-soluble cationic conjugated oligopyrene derivative, oligo(N^1^,N^1^,N^1^,N^4^,N^4^,N^4^-hexamethyl-2-(4-(pyren-1-yl) butane-1,4-diaminium bromide) (OHPBDB), was synthesized by a combination of chemical and electrochemical synthesis techniques. Each oligomer chain has five pyrene derivative repeating units and brings 10 positive charges. OHPBDB showed high and rapid fluorescence quenching in aqueous media upon addition of trace amounts of single-stranded (ss) and double-stranded (ds) DNA. The Stern-Volmer constants for ss- and ds-DNA were measured to be as high as 1.3 × 10^8^ mol^−1^·L and 1.2 × 10^8^ mol^−1^·L, respectively. On the other hand, distinct fluorescence enhancement of OHPBDB upon addition of large amount of ss-DNA or ds-DNA was observed. Furthermore, ss-DNA showed much stronger fluorescence enhancement than that of ds-DNA, thus yielding a clear and simple signal useful for the discrimination between ss- and ds-DNA in aqueous media.

## 1. Introduction

It has been proven that a fluorescent conjugated polymer is far more sensitive than the corresponding fluorescent monomer probe for detecting trace chemicals and biological analytes. These properties arise from their exceptional inter- or intra-chain energy transfer and relatively strong light-harvesting ability [1-3], due to the delocalization of electrons along the conjugated backbones. The most characteristic features of these polymers are their low reduction potentials and low-lying Lowest Unoccupied Molecular Orbitals (LUMOs). As a result, the electric, optical and electrochemical properties of these polymers can be changed by slightly altering their chemical structures and can be strongly affected by even a relatively small and momentary perturbance induced by the presence of a trace analyte. Among the numerous reported probe molecules, cationic water-soluble conjugated polymers (CCPs) are of particular significance for the design of ultra-sensitive DNA sensors because of their enhanced interfacial binding to these biological species. Water-soluble CCPs usually contain hydrophilic side chains bearing positively charged groups. Typical examples are the poly(fluorene) and poly(3-alkoxy-4-methylthiophene) derivatives reported by the Bazan [3-11] and Leclerc [12,13] groups. In principle, the biosensors based on CCPs show promising optical properties in three aspects: first, they exhibit different electrostatic interactions and conformational structures upon binding to different target DNA sequences [12]. Second, they display strong light harvesting ability that can induce the optical amplification of a dye-labeled probe signal [3]. Third, they are able to detect target DNA in aqueous solutions at a very low concentration by measuring their fluorescence superquenching signals [14]. Although these established advantages in the previous methods are well known, they still show at least two drawbacks. First, for the third case, it is believed that one of the main reasons for the superquenching arises from the π–π interaction among the aromatic segments, which is driven by a combination of electrostatic and hydrophobic attraction between cationic conjugated polymers and negatively charged DNA. However, almost all the CCPs reported previously contain small aromatic units consisting of 5- or 6-membered ring structures, leading to an inefficient backbone π–π stacking and thus reducing their quenching efficiency. We speculate that the superquenching efficiency of the water-soluble conjugated polymers might be increased by introducing dense aromatic units into their backbones. Nevertheless, only a limited number of conjugated polymers or oligomers with dense aromatic units have been synthesized, and few of them have been applied for ultra DNA detection. Second, all the three methods are widely employed for sensing single nucleotide polymorphism (SNP), but they are often inadequate to directly discriminate between single-stranded DNA (ss-DNA) and double-stranded DNA (ds-DNA) by sensing the probe signal since they specifically require harsh denaturation conditions for hybridization to a complementary single-stranded DNA [15-23]. It is known that DNA damage has been determined to be responsible for the induction of cell lethality, mutagenesis and carcinogenesis [24]. In some cases, this damage will render a weakened polymer structure at the site of the attack (alkali labile site (ALS)) and give rise to strand breaks or cleavage [25,26]. Therefore, it is significant for discrimination between ss-DNA and ds-DNA. The conventional technique for this discrimination, using reversed-phase high-performance liquid chromatography (RP-HPLC), is expensive and time-consuming [27]. Several optical sensors were also developed for this purpose. One method relies on measuring the lifetime changes of the probe molecule upon binding to DNA [28]; another depends on measuring the changes in fluorescence intensity of the small molecular probe upon intercalating into DNA [23]. However, most of these available probes cannot efficiently differentiate between ds-DNA and ss-DNA [28]. Although studies on the size-specific interactions between CCP and ss- or ds-DNA by measuring its optical responses in the presence of the chromophore-labeled DNA probe have been reported [3], there is scant literature concerning the discrimination between ss-DNA and ds-DNA using a label-free DNA assay combining with CCP as a direct signal reporter.

In this paper, we report the sensing and discriminating of ss-DNA and ds-DNA by the use of a unique water soluble conjugated oligopyrene derivate, OHPBDB, as the probe molecule. This oligomer was selected because of its good solubility in aqueous media and its bulky aromatic backbone. The experimental results indicated that the fluorescence quenching rate constants of OHPBDB for ss-DNA and ds-DNA measured at low DNA concentration were nearly identical with the values of 1.3 × 10^8^ mol^−1^·L and 1.2 × 10^8^ mol^−1^·L, respectively, which are much higher than those of using the corresponding monomer, HPBDB. However, at high DNA concentrations, OHPBDB was found to display a much stronger concentration-dependent fluorescence recovery upon ss-DNA as compared with ds-DNA. Thus, based on the large difference in the fluorescence enhancement of OHPBDB aqueous solution enables to distinguish ds-DNA from ss-DNA. This technique has potential application in quantifying single strand or double strand breaks in DNA [22,23].

## 2. Results and Discussion

### 2.1. Absorption and fluorescence properties of OHPBDB and HPBDB

**Scheme 1.**
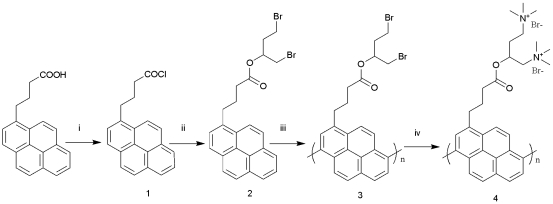
Synthesis of OHPBDB. *Reagents and conditions*: (i) Oxalyl chloride (3 equiv.), CH_2_Cl_2_, DMF(cat.), 18 °C, 98%; (ii) 1,4-dibromo-2-butanol (1.5 equiv.), CH_2_Cl_2_, DMF (cat.), K_2_CO_3_, 18 °C, 94%; (iii) 0.75 V vs Ag/AgCl, BFEE, DMF, room temperature, 36%; (iv) N(CH_3_)_3_, THF, 18 °C, 82.2%.

The structures of the water-soluble cationic conjugated oligopyrene derivative (OHPBDB) and its corresponding monomer (HPBDB) are shown in Schemes 1 and S1 (in the supporting information).

**Figure 1.**
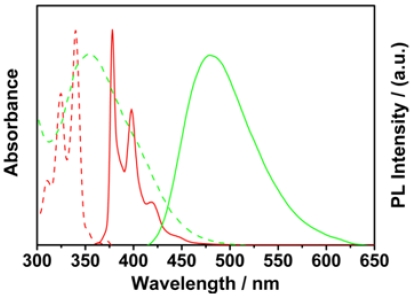
Normalized absorption (dashed lines) and fluorescence spectra (solid lines) of the aqueous solutions of HPBDB (red) with a concentration of 3.3 × 10^−5^ mol·L^−1^ and OHPBDB (green) with a repeat unit concentration of 3.3 × 10^−5^ mol·L^−1^.

Figure 1 displays the absorption and fluorescence spectra of OHPBDB and HPBDB aqueous solutions. The absorption and fluorescence spectra of HPBDB are similar to those of 2-(4-(1-pyrenyl)butanoyloxy)ethyltrimethylammonium bromide (OPBEAB), previously reported by us [29]. The three strong sharp absorption bands at 308, 319 and 335 nm for HPBDB are ascribed to the three vibronic sub-bands of S_0_→S_2_ transitions of the single electron transfer in the pyrene ring [30]. The weak absorption band at a wavelength of 375 nm is assigned to the S_0_ → S_1_ vibronic band of the single electron transfer. In contrast, OHPBDB exhibits only one broad absorption at 354 nm across a wide wavelength range, which is attributed to the π– π * transition localized on the conjugated backbone. The absorption position at the longest wavelength is red shifted by about 19 nm in comparison to that of HPBDB (335 nm). When excited at 340 nm the HPBDB fluorescence spectrum exhibits well-defined vibronic structure with three peaks at 378, 398, 418 nm, while the OHPBDB emission displays only a broad and structure-less peak centered at 480 nm when excited at 340 nm, which shows a distinct red-shift (by 102 nm) compared to that of HPBDB (378 nm). OHPBDB in water is less emissive, with a fluorescence quantum yield of 0.06 in comparison to HPBDB which has a corresponding value of 0.97. This is ascribed to the OHPBDB chain aggregation, which in turn leads to the CCP-CCP self-quenching. However, the Φ value of OHPBDB is twice that of OPBEAB. This is mainly due to that the charge density per repeat unit of the former is twice that of the latter. Thus, the stronger electrostatic repulsion between OHPBDB chains resulted in a less tendency of CCP aggregation in water. The strong infrared absorption at around 815 cm^−1^ for OHPBDB revealed that it is made up of αC-αC linked pyrene derivative units. 3 – 6, 3 – 8 links are preponderant due to their smaller structural steric hindrances and lower oxidation potential of 0.75 V (vs. Ag/AgCl) [29]. The molecular structure of 3–8 coupled oligopyrene derivative is illustrated in Scheme 1 for simplification.

### 2.2. Fluorescence quenching of OHPBDB upon ss-DNA and ds-DNA

**Figure 2.**
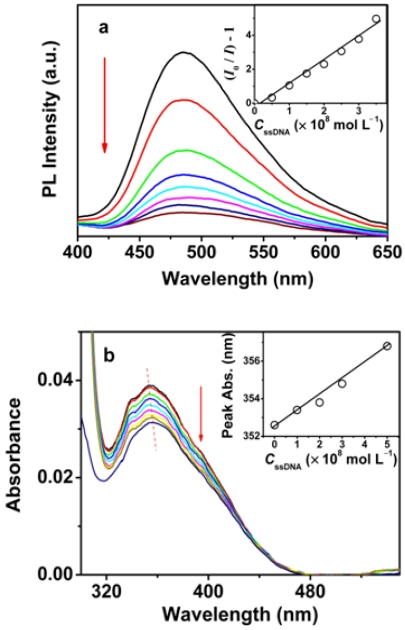
Fluorescence (a) and absorption (b) spectra of OHPBDB as a function of *C*_ss-DNA_ in Tris-HCl buffer (10 mmol·L^−1^; pH = 7.4); *C*_OHPBDB_ = 3.34 × 10^−6^ mol·L^−1^; (a) *C*_ss-DNA_ = 0, 5 × 10^−9^, 1.0 × 10^−8^, 1.5 × 10^−8^, 2.0 × 10^−8^, 2.5 × 10^−8^, 3.0 × 10^−8^, 3.5 × 10^−8^ mol·L^−1^; (b) *C*_ss-DNA_ = 0, 5 × 10^−9^, 1.0 × 10^−8^, 1.5 × 10^−8^, 2.0 × 10^−8^, 2.5 × 10^−8^, 3.0 × 10^−8^, 3.5 × 10^−8^, 5.0 × 10^−8^ mol·L^−1^; *λ*_ex_ = 340 nm. The direction of the arrow indicates the increase of ss-DNA concentration. Note that *C*_OHPBDB_ and *C*_ss-DNA_ in all figures represent the concentrations of OHPBDB and ss-DNA respectively.

For the detection of DNA, fluorescence titrations were performed to monitor the fluorescence change due to the binding of DNA to OHPBDB. As shown in Figure 2(a), the OHPBDB fluorescence emission at the excitation wavelength of 340 nm decreased drastically upon the successive addition of aliquots of ss-DNA solution. However, the absorption maximum of OHPBDB aqueous solution, as shown in Figure 2(b), is slightly red shifted about 3 nm from 354 to 357 nm as the concentration of ss-DNA (*C*_ss-DNA_) increased from 0 to 3.3 × 10^−5^ mol·L^−1^. At *C*_ss-DNA_ = 1.0 ×10^−8^ mol·L^−1^, the fluorescence quenching efficiency of OHPBDB upon ss-DNA, (*I*_o_–*I*)/*I*_o_ (*I* and *I*_o_ are the fluorescence intensity of the OHPBDB aqueous solution with and without ss-DNA, respectively), was measured to be as high as approximately 50%. Based on the correlation between *I* value and *C*_ss-DNA_ as shown in Figure 2(a), the concentration of ss-DNA can be detected in the range of 5 × 10^−9^ to 3.5×10^−8^ mol·L^−1^. The detection limit is extremely low to be about 5 × 10^−9^ mol·L^−1^ and this value is lower than that using a dye end-labeled peptide nucleic acid (PNA) reported previously [5].

The quantitative description of the quenching process can be represented by the Stern-Volmer equations:

(1) $$I_{0}/I=1+K_{\text{sv}}\left[\text{Q}\right]$$

where *K*_sv_ is the Stern-Volmer constants, and [Q] is the concentration of analyte, here, it refers to *C*_ss-DNA_. The value of *K*_sv_ describes the quenching efficiency by the quencher. In the case of purely static quenching, *K*_sv_ equals the association constant of the bound complex. It is clear from the inset of Figure 2(a) that the Stern-Volmer plot exhibits a straight line at all concentrations of ss-DNA. The *K*_sv_ value was calculated to be 1.3 × 10^8^ mol·L^−1^, which is approximately four orders of magnitude larger than that of using a small fluorescent molecule [32]. On the other hand, the extremely large *K*_sv_ indicates that the static quenching is the predominant factor in the OHPBDB/ss-DNA system [33].

The fluorescence quenching of OHPBDB might be derived from various quenching species [29]. Its real time and highly sensitive fluorescence responses of OHPBDB upon ss-DNA are mainly due to following factors. First, the fluorescence superquenching can be induced by the molecular wire effect which entails sensory signal amplification [34]. This effect is resulted from the photoinduced charge transfer in OHPBDB/ss-DNA complex between contacted OHPBDB and ss-DNA chains. It can also be induced from the photoinduced charge transfer from OHPBDB chain on the outer surface of the OHPBDB/ss-DNA aggregates to ss-DNA. The charge transfer in this pathway might be mediated through the favorable π–π stacking between the large delocalized hydrophobic backbones of OHPBDB. Second, the excited OHPBDB state can be quenched by ss-DNA in a static process by forming the nonradiative bound complex in its ground state. The occurrence of this quenching is due to the existence of the closely hydrophobic contacts between large OHPBDB backbones and ss-DNA residues. Third, OHPBDB chains showed self-quenching. This is possible due to that the normalized OHPBDB emission is partially overlapped with its absorption as shown in Figure 1. It should be noted that the molecular surface area of a pyrene unit is estimated to be 20.1 Å^2^ by theoretical calculation [35]. This value is approximately twice that of the fluorene unit of oligofluorene and four times that of thiophene unit of poly(3-alkoxy-4-methylthiophene). Therefore, OHPBDB displayed a more efficient quenching by a strong π–π electron interactions among OHPBDB chains for all the three quenching cases described above.

**Figure 3.**
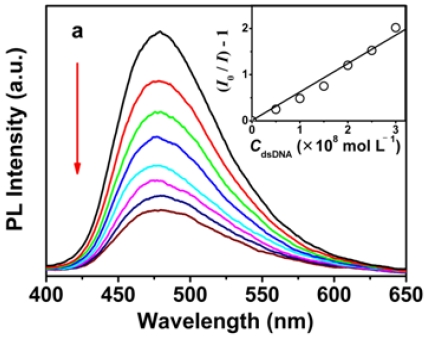

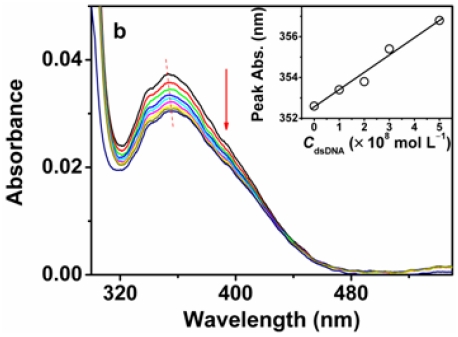
Fluorescence (a) and absorption spectra (b) of the aqueous solution of OHPBDB as a function of *C*_ds-DNA_ in Tris-HCl buffer (10 mmol·L^−1^; pH = 7.4); (a) *C*_ds-DNA_ = 0, 5 × ^10−9^, 1.0 × 10^−8^, 1.5 × 10^−8^, 2.0 × 10^−8^, 2.5 × 10^−8^, 3.0 × 10^−8^, 3.5 × 10^−8^ mol·L^−1^; *C*_OHPBDB_ = 3.34 × 10^−6^ mol·L^−1^; (b) *C*_ds-DNA_ = 0, 5 × 10^−9^, 1.0 × 10^−8^, 1.5 × 10^−8^, 2.0 × 10^−8^, 2.5 × 10^−8^, 3.0 × 10^−8^, 3.5 × 10^−8^, 5.0 × 10^−8^ mol·L^−1^; *λ*_max_ = 340 nm. The direction of the arrow indicates the increase of ds-DNA concentration. Note that *C*_ds-DNA_ represents the concentration of ds-DNA.

To get a deeper insight into the efficient quenching of OHPBDB by the oligonucleotides, we also studied the fluorescence quenching of OHPBDB solution upon addition of ds-DNA (ds-DNA in the assay contains a same strand of ss-DNA described above and a complementary strand: 5′-AGTAACTCAAGT-3′. The fluorescence quenching and absorption of OHPBDB upon trace addition of ds-DNA are shown in Figures 3(a) and (b).

Neither of the spectra display any difference in features compared to those of the OHPBDB/ss-DNA solution. The *K*_sv_ measured for the case of ds-DNA is 1.2 ×10^8^ mol·L^−1^, which is nearly identical to that of ss-DNA. This phenomenon indicates that the binding abilities of OHPBDB to ss-DNA and ds-DNA are almost the same. However, the available negative charge coming from the phosphate groups of ds-DNA is about twice that of ss-DNA. Therefore, the electrostatic interaction force between OHPBDB and ds-DNA should be stronger than that between OHPBDB and ss-DNA. The explanation of the spectral result described above is that the ss-DNA chain has larger exposed hydrophobic surface than that of ds-DNA and its stronger π–π interaction with bulky pyrene units of OHPBDB backbone partially compensates its relatively weaker electrostatic interaction [3].

Control experiments showed that HPBDB, the monomer of OHPBDB, exhibited a *K*_sv_ of 4.2 × 10^6^ mol·L^−1^ for ss-DNA and that for ds-DNA was measured to be 5.8 × 10^6^ mol·L^−1^. These values are much lower than those of OHPBDB, indicating the fluorescence of the oligomer can be quenched more efficiently than that of its monomer upon ss-DNA or ds-DNA.

### 2.3. Fluorescence enhancement of OHPBDB upon ss-DNA and ds-DNA

**Figure 4.**
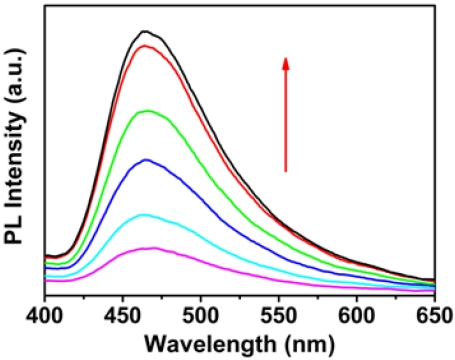
Fluorescence spectra of OHPBDB as a function of *C*_ss-DNA_ in Tris-HCl buffer (10 mmol·L^−1^; pH = 7.4); *C*_OHPBDB_ = 3.34 × 10^−5^ mol·L^−1^; *C*_ss-DNA_ = 8.33 × 10^−7^, 1.67 × 10^−6^, 3.33 × 10^−5^, 8.33 × 10^−5^, 1.67 × 10^−5^, 3.33 × 10^−4^ mol·L^−1^ *λ*_ex_ = 365 nm. The direction of the arrow indicates the increase of ds-DNA concentration.

In general, the phases of multiple possible quenching species as well as their related optical properties depend on the concentrations of CCP or/and DNA. Therefore, we studied the fluorescence spectra of OHPBDB upon addition of ss-DNA, ds-DNA and ss-DNA/ds-DNA mixture in a high concentration region (8.33 × 10^−7^ to 1.67 × 10^−5^ mol·L^−1^). As shown in Figure 4, the fluorescence intensity of OHPBDB solution increased dramatically as the concentration of ss-DNA increased from 8.33 × 10^−7^ to about 1.67 × 10^−5^ mol·L^−1^. The wavelength of the emission maximum is blue shifted for 15 nm, from 480 nm (Figure 2) to 465 nm (Figure 4). This change corresponds to an abrupt reduction in the polarity within the microenvironment surrounding OHPBDB molecules in the OHPBDB/ss-DNA complex [29].

**Figure 5.**
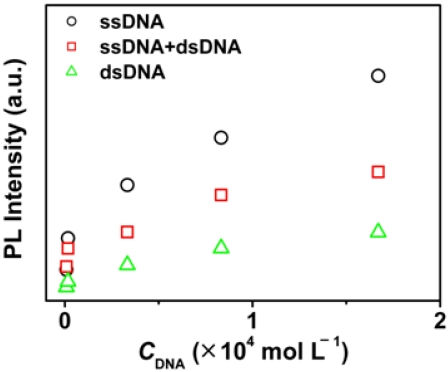
Fluorescence enhancement of OHPBDB as a function of the concentration of ss-DNA, ds-DNA, and ss-DNA and ds-DNA mixture (8.33 × 10^−7^, 1.67 × 10^−6^, 3.33 × 10^−5^, 8.33 × 10^−5^, 1.67 × 10^−5^ and 3.33 × 10^−4^ mol·L^−1^, respectively) in Tris-HCl buffer (10 mmol·L^−1^; pH = 7.4). *C*_OHPBDB_ = 3.34 × 10^−5^ mol·L^−1^; *λ*_ex_ = 365 nm.

The nature of this spectral blue shift is due to the breakup of the former loose aggregates of OHPBDB (at low *C*_ss-DNA_) induced by the excessive addition of ss-DNA [29]. Similar phenomenon was also observed in the case of using ds-DNA or the mixture of ss- and ds-DNA (Figure 5).

However, at a given DNA concentration, the fluorescence enhancement induced by ss-DNA is approximately 2.7 times that caused by ds-DNA; meanwhile it is approximately 1.3 times that by using a mixture of ss-DNA and ds-DNA. This phenomenon can be explained as follows: at very low DNA concentration, OHPBDB/DNA complexes may have structures which contain many aggregated oligomers surrounding a single DNA chain [3]. It should be noted that these aggregates are loose dynamic structures with a range of sizes [3]. Therefore, in the case of addition a large amount of ss-DNA, the free ss-DNA chains in solution can continuously peel off the loosely bound OHPBDB molecules from OHPBDB/ss-DNA complexes. Finally, due to the hydrophobic attraction between ss-DNA chains, some structures which contain many ss-DNA chains surrounding a single or several oligomer molecules could be spontaneously formed. If the former structure did exist, it must meet another key factor that both size and the number of charges of OHPBDB match those of ss-DNA for the assay. As described above, the oligomer having five repeat units bears 10 positive charges. Its chain length is calculated to be approximately 3.3 nm if we regard it as a linearly rigid rod structure shown in Scheme 1. Whereas, the size of 12 base DNA was estimated to be 3.6 nm based on the mean size of a single base, ˜ 3Å [37]. Thus, these comparable sizes and charge numbers of OHPBDB and ss-DNA chains are favorable for the formation of the complex containing only one oligomer chain. This newly formed structure reduces interchain quenching since it contains only single oligomer chain [29]. It also reduces the fluorescence quenching by water by preventing the nonoradiative process through reducing the polarity surrounding the oligomer surface [29]. Therefore, various quenching species which compete with emissive intrachain exciton relaxation in OHPBDB/ss-DNA system is greatly restricted in the solutions with high *C*_ss-DNA_. Accordingly, a drastic enhancement of OHPBDB emission was observed in these cases.

**Figure 6.**
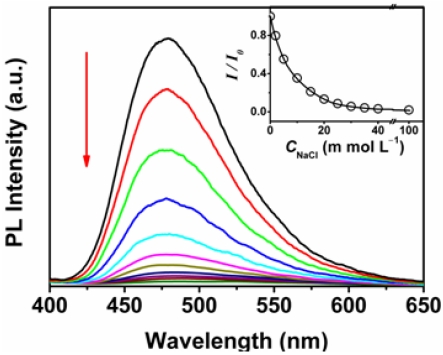
Fluorescence spectra of the aqueous solution of OHPBDB as a function of NaCl concentration, *C*_NaCl_ in Tris-HCl buffer (10 mmol·L^−1^; pH = 7.4); *C*_NaCl_ = 0, 2, 5, 10, 15, 20, 25, 30, 35, 40, 100 mol·L^−1^; *λ*_max_ = 340 nm. The direction of the arrow indicates the increase of NaCl concentration.

Upon addition of a large amount of ds-DNA into OHPBDB solution, free ds-DNA can also initially peel off the loosely bound OHPBDB molecules from OHPBDB/ds-DNA complex. However, free ds-DNA is a helical structure in water having a hydrophobic core in the center and negatively phosphate groups outside its cylindric surface. This structure prevents efficient hydrophobic interaction between OHPBDB and ds-DNA. Furthermore, the number of charges coming from an OHPBDB chain is less than half that of a ds-DNA chain. Therefore, a ds-DNA chain has the propensity to associate with several OHPBDB molecules by forming a complex that is strongly linked by ionic bonds. This new complex reduces the fluorescence emission by CCP⋯CCP self-quenching [3]. Additionally, the new complexes of which the charges are neutralized would have a structure that bulky aromatic backbones of OHPBDB are more exposed to external media than that of a OHPBDB/ss-DNA complex. Thus, they can further aggregate each other to form an even larger aggregate through the favorable π–π stacking effect (the out surface of ds-DNA is hydrophilic). This explanation has been confirmed by the high sensitivity of the OHPBDB fluorescence emission upon ionic strength in solution as shown in Figure 6. Based on these reasons, OHPBDB showed a stronger fluorescence enhancement upon addition of a given concentration of ss-DNA as compared to ds-DNA or the mixture of ss-DNA and ds-DNA. On the other hand, one cannot observe a fully emission recovery from its original intensity of the oligomer by addition sufficient amount of ss-DNA (e.g. *C*_ss-DNA_ = 1.67 × 10^−5^ mol·L^−1^). The reason is that the system still containing OHPBDB/ss-DNA complexes.

Control experiments using HPBDB as the probe produced no fluorescence enhancement even when *C*_ss-DNA_ and *C*_ds-DNA_ are as high as 3.3 × 10^−5^ mol·L^−1^. This is possibly due to that HPBDB bears only two charges and has a smaller molecular size; both of them do not match those of DNA.

## 3. Experimental Section

### 3.1. Materials

Oxalyl chloride and trimethylamine were purchased from Beijing Chem. Reagent Ltd (Beijing, P.R. China). Pyrenebutyric acid (PBA) and 1,4-dibromo-2-butanol were purchased from ACROS. All the chemicals described above were of analytical reagent grade and used directly and Milli-Q water was used throughout the work. Boron trifluoride diethyl etherate (BFEE) was a product of Changyang Chemical Plant (Beijing, P.R. China) and purified by distillation before use. The single and double stranded DNAs were purchased from TaKaRa Biotechnology Co., Ltd (Dalian, P.R. China). All stock solutions of DNA were prepared by dissolving certain amount of solid DNA in water overnight and stored in a refrigeator at 4 °C in the dark for less than a week, and their concentrations were determined by absorption spectrometry, using the absorptivity ε_260_ = 6,600 mol^−1^·cm^−1^. Purity of DNA was checked by measuring the ratio of the absorbance at 260 nm to that at 280 nm. The solution gave a ratio of A_260_/A_280_ > 1.8, indicating that DNA was sufficiently free from protein [38]. All the reagents described above were used as received unless otherwise noted. Oligo(N^1^,N^1^,N^1^,N^4^,N^4^,N^4^-hexamethyl-2-(4-(pyren-1-yl)butane-1,4-diaminium bromide) (**4**, OHPBDB) was synthesized following Scheme 1. Synthesis of N^1^,N^1^,N^1^,N^4^,N^4^,N^4^-hexamethyl-2-(4-(pyren-1-yl) butane-1,4-diaminium bromide (**5**, HPBDB) was similar to that of OHPBDB. Detailed procedures for synthesizing compounds 1-5 are provided in the supporting information.

### 3.2. Characterizations

NMR (^1^H- and ^13^C-) spectra were recorded on a JNM-ECA300 NMR spectrometer (300 MHz; JEOL, Tokyo, Japan) and chemical shifts are reported in parts per million relative to TMS in proton spectra. All ^13^C-NMR chemical shifts were recorded as decoupled spectra. FT-IR spectra were recorded on a Magna 560-IR spectrometer (Nicolet, Madison, WI, USA) equipped with a KBr beam splitter and a DTGS detector. For each spectrum, 10 scans were acquired with 1-cm^−1^ resolution over a wavelength range of 4,000–400 cm^−1^. The samples were pressed into KBr pellets. High-resolution mass spectra were obtained with Esquire-LC 00136 mass spectrometer (Bruker Daltonics, Bremen, Germany) using sector double focus and electron impact source with an ionizing voltage of 70 V. UV measurements were taken on a U-3010 spectrophotometer (Hitachi High-Technologies Corp., Japan). Elemental analysis was performed by a CE-440 elemental analyzer (EAI Corp., USA). The molecular weight and polydispersity of compound 3 (ODBBPB) were measured by an Agilent 1,100 LC gel permeation chromatography (Agilent Technologies Inc., Palo Alto, CA, USA) using polystyrene as standard and tetrahydrofuran as the solvent.

### 3.3. Fluorescence and DNA sensing studies

Fluorescence spectra were recorded with a Perkin–Elmer luminescence spectrometer (model LS 55, 75-W xenon lamp) at 25 °C. The quantum yield (Φ) measurements for compounds **4** and **5** were performed in aqueous solutions [39], and the corrected fluorescence spectra were used for calculation. The excitation wavelength was 330 nm and quinine sulfate monohydrate was employed as a standard (Φ = 0.55; in 50 mmol·L^−1^ H_2_SO_4_ aqueous solution).

The single stranded oligonucleotide (ss-DNA) chosen for this study has 12 bases with a sequence of 5′-ACTTGAGTTACT-3′. The double stranded oligonucleotide (ds-DNA) has a same strand of ss-DNA and a complementary strand (5′-AGTAACTCAAGT-3′). DNA binding studies were carried out in 10 mmol·L^−1^ Tris-HCl buffer (pH = 7.4) containing 2 mmol·L^−1^ NaCl since it has a distinct role in the regulation of the DNA-DNA interaction in water. In all the tests, the concentration units of ss-DNA or ds-DNA were given in terms of oligonucleotide strands throughout the work.

## 4. Conclusions

A water-soluble cationic conjugated oligopyrene derivative, OHPBDB, has been successfullly synthesized by the combination of chemical and electrochemical reactions. The large dense aromatic repeating units of this oligomer provide it with strong π–π stacking interactions among its chains in aqueous media. This unique strong hydrophobic interaction force effectively compensates the inherent weaker electrostatic interaction in the OHPBDB/ss-DNA aggregates than that in OHPBDB/ds-DNA aggregates. As a result, at very low DNA concentration, OHPBDB aqueous solutions exhibited similar fluorescence quenching efficiencies upon addition of ss-DNA or ds-DNA. On the other hand, at high DNA concentrations, OHPBDB showed distinct fluorescence enhancement with increasing ss-DNA and ds-DNA concentrations, mainly due to the hydrophobic collapse of the OHPBDB aggregates. This difference in the emission changes is due to the presence of the stronger electrostatic attraction in the OHPBDB /ds-DNA complex as compared to that in OHPBDB/ss-DNA system. Therefore, the fluorescence enhancement selectivity is shown between ss-DNA and ds-DNA assays at high DNA concentrations.
